# CT virtual endoscopy and 3D stereoscopic visualisation in the evaluation of coronary stenting

**DOI:** 10.2349/biij.5.4.e22

**Published:** 2009-10-01

**Authors:** Z Sun, Lawrence-Brown

**Affiliations:** 1 Discipline of Medical Imaging, Department of Imaging and Applied Physics, Curtin University of Technology, Perth, Australia; 2 School of Public Health, Curtin University of Technology, Perth, Australia

**Keywords:** Coronary artery disease, virtual endoscopy, coronary stent, three-dimensional visualization, stereoscopic visualisation

## Abstract

The aim of this case report is to present the additional value provided by CT virtual endoscopy and 3D stereoscopic visualisation when compared with 2D visualisations in the assessment of coronary stenting. A 64-year old patient was treated with left coronary stenting 8 years ago and recently followed up with multidetector row CT angiography. An in-stent restenosis of the left coronary artery was suspected based on 2D axial and multiplanar reformatted images. 3D virtual endoscopy was generated to demonstrate the smooth intraluminal surface of coronary artery wall, and there was no evidence of restenosis or intraluminal irregularity. Virtual fly-through of the coronary artery was produced to examine the entire length of the coronary artery with the aim of demonstrating the intraluminal changes following placement of the coronary stent. In addition, stereoscopic views were generated to show the relationship between coronary artery branches and the coronary stent. In comparison with traditional 2D visualisations, virtual endoscopy was useful for assessment of the intraluminal appearance of the coronary artery wall following coronary stent implantation, while stereoscopic visualisation improved observers’ understanding of the complex cardiac structures. Thus, both methods could be used as a complementary tool in cardiac imaging.

## INTRODUCTION

In recent years, coronary artery disease has been increasingly treated by coronary stent placement. Although stent implantation has been shown to greatly reduce restenosis after balloon angioplasty [1, 2], in-stent restenosis can occur in 20-35% of patients for bare metal stents [3, 4], and 5-10% for drug-eluting stents [3, 4]. Conventional catheter-based coronary angiography remains the gold standard technique for detection of in-stent restenosis. However, coronary angiography has limitations due to its invasiveness and associated potential risks of morbidity and mortality. Given the high number of patients who receive coronary stents yearly, a non-invasive imaging technique for follow-up of coronary stenting and detection of in-stent restenosis will be clinically important and beneficial.

Currently multi-detector row computed tomography (MDCT) is increasingly used for non-invasive imaging of coronary artery disease and has been reported to have a high diagnostic accuracy in the detection of coronary artery stenosis and in-stent restenosis, especially with the development of the latest 64-detector row CT scanners [5-7]. Nearly isotropic volume data are acquired with 64 MDCT scanners which allow generation of a series of 3D reconstructions of the coronary artery and stents. Of these reconstructions, multiplanar reformation (including curved reformation), maximum-intensity projection and volume rendering are commonly used in the visualisation of coronary stenting. In contrast, two other types of 3D visualisations including virtual endoscopy and stereoscopic views offer additional information when compared to 2D reconstructions. Virtual endoscopy offers unique intraluminal views of the artery lumen and stent structures [8-10], while stereoscopic visualisation provides a realistic 3D view which allows demonstration of complex anatomic structures [11, 12].

In this case report, we presented our experience of using virtual endoscopy and 3D stereoscopic views for visualisation of the coronary artery and coronary stent in a patient treated with coronary stenting. The aim of the case report was to identify the potential value of these 3D visualisations in demonstration of complicated cardiac structures when compared to conventional 2D visualisations.

## PATIENT HISTORY>

A 64-year old man presented with exertional angina in 1998 and was investigated with an exercise ECG, which showed weakly positive results. Thallium scans were negative for differential differences in left ventricular coronary artery flow. Persistent angina was further investigated in 1999 with coronary angiography and showed a 70% stenosis at the origin of the left main coronary artery. This was treated with a Left Internal Mammary Artery (LIMA) bypass to the descending and long saphenous Vein Bypass (LSV) to the circumflex coronary arteries. Angina persisted and repeated angiography in 2000 showed that the LIMA bypass was not patent and the stenosis had progressed. The origin of the left main coronary artery was treated with a bare stent and dilated to 4 mm. In 2008 further investigation was requested for travel insurance and the CT angiography was performed to avoid invasive investigation.

## IMAGING GENERATION AND VISUALISATION

### Conventional 2D reconstructions

**Figure 1:**
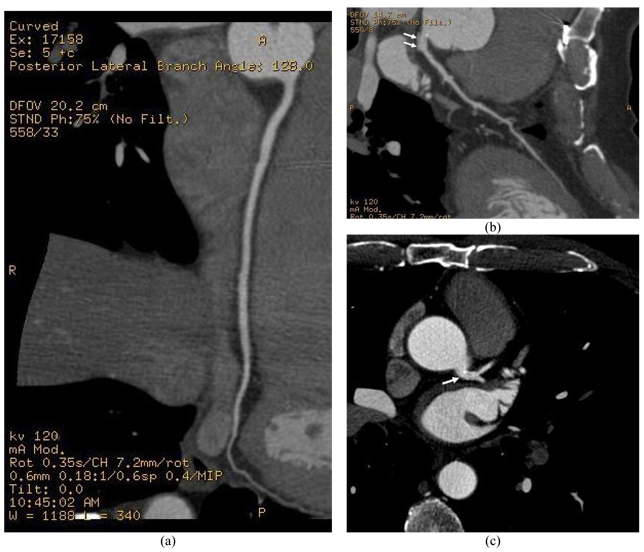
(a) Is a multiplanar reformatted image of the right coronary artery, while (b) and (c) show the left coronary artery with coronary stent implanted (arrows) at the ostium of left main stem, visualised on multiplanar reformatted and 2D axial views.

Coronary CT angiography was performed using a 64-detector row CT scanner (64x0.625, Lightspeed VCT, GE Medical Systems) with a section thickness of 0.625 mm and reconstruction interval of 0.4 mm. Curved multiplanar reformatted images were generated at the CT scanner workstation. Figure 1 shows the multiplanar views of right and left coronary arteries. Left coronary stent was confirmed to be patent on CT images, but restenosis was suspected at the left coronary artery based on the multiplanar reformatted images.

### Virtual endoscopy and 3D stereoscopic visualisation

**Figure 2:**
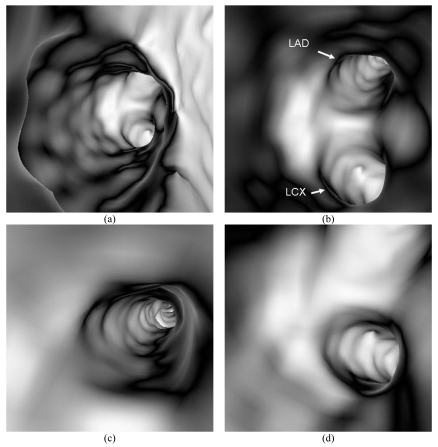
(a) Virtual endoscopy views of the ostium, and (b) proximal segment of left coronary artery, (c) left anterior descending (LAD), and (d) left circumflex (LCX). The internal wall of these artery branches looks smooth on virtual endoscopy images with no sign of intraluminal irregularity.

The original DICOM (digital imaging and communication in medicine) data was transferred to a separate workstation equipped with Analyze V 7.0 (AnalyzeDirect, Inc., Lexana, KS, USA) for generation of virtual endoscopic and stereoscopic images. Generation of virtual endoscopic images was performed with a CT thresholding technique, which has been described before [8]. Intraluminal views of the proximal, middle and distal left stented coronary artery were produced with no evidence of restenosis, as shown in figure 2. The lumen of the left coronary artery remained smooth and there was no sign of any endothelial thickness or endoluminal irregularity caused by the coronary stent.

In addition to the static virtual endoscopy visualisations, we also produced dynamic fly-through of the coronary arteries with the aim of demonstrating the entire coronary arteries. This was performed by setting a number of virtual cameras along the path of the coronary arteries. A fly-through of the left coronary artery including left anterior descending and left circumflex branch confirmed the findings of static virtual endoscopic visualisations, which showed the smooth appearance of coronary lumen.

**Figure 3:**
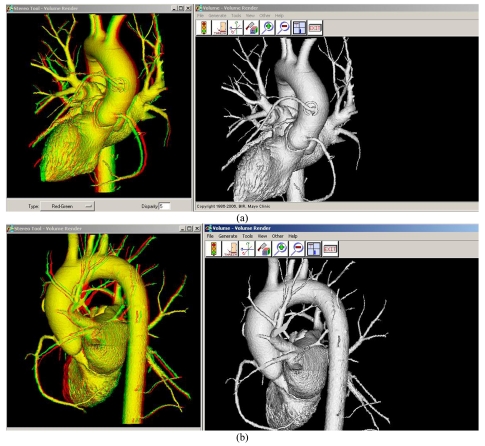
Stereoscopic visualisation of right coronary artery and left coronary stent as well as bypass graft compared with conventional surface rendered visualisation, observed in (a) frontal view and (b) lateral view. (red/blue glasses are required to appreciate the stereoscopic effect).

A stereoscopic pair of images consists of two projections of the same 3D object acquired from two slightly different viewing angles. The pair of stereoscopic images is displayed so that only the left eye sees the left projection and only the right eye sees the right projection. As a result, the observer is able to reconstruct and appreciate the 3D object mentally including the depth dimension. In our study, the reviewers used red/blue glasses for the stereoscopic display of the cardiac anatomic structures as shown in Figure 3. This allows the reviewers to better appreciate the complex anatomic structures when compared with 2D visualisations.

## DISCUSSION AND CONCLUSION

MDCT has been increasingly used for the diagnosis of coronary artery disease and follow-up of coronary stenting due to its improved spatial and temporal resolution, especially with the advancement of 64-detector row scanners [5-7]. In addition to axial CT images, a number of reconstructed visualisations are essential and play an important role in the demonstration of complex coronary arteries. Of these reconstructions, curved multiplanar reformation is the most commonly used tool for visualisation of the coronary artery tree, complemented by maximum-intensity projection or volume rendering. However, there are still limitations with curved multiplanar reformation as it does not provide intraluminal information about the coronary artery or coronary stent. This can be easily resolved with virtual endoscopy and virtual fly-through visualisations, as reported previously in other endovascular applications [8-10].

Our results in this case study demonstrated the superiority of virtual endoscopy with intraluminal views over conventional extraluminal visualisations. This is manifested by visualisation of the smooth intraluminal surface in both stented and normal coronary artery lumen. In this case, virtual endoscopy excludes the presence of coronary in-stent re-stenosis or any stent-related changes in the coronary artery wall.

We also included stereoscopic visualisation in this case and our results showed that it offers better understanding of the complex cardiac structures. Our previous study confirmed that stereoscopic viewing provides additional information regarding any distortions of the fenestrated stents compared with conventional 2D visualisations [12]. Thus, stereoscopic visualisation was recommended as a complementary tool for follow-up of fenestrated stent grafting. Similarly, this visualisation tool can be used as complementary to 2D views in the follow-up of coronary stenting, although this needs verification in a large cohort.

Drug-eluting stents are increasingly being used in clinical practice with the aim of preventing in-stent restenosis, and research has shown the decreased incidence of stent re-stenosis when compared to bare metal stents [13, 14]. However, there is growing concern that delayed endothelisation and incomplete neointimal healing might lead to adverse cardiac outcomes and death as a result of late or very late stent thrombosis [15, 16]. The inevitable arterial injury due to balloon deployment of a stent coupled with the presence of a metallic foreign body can cause inflammatory and proliferative responses [17]. The development of in-stent restenosis after implantation is due to the neointimal hyperplasia around the stent in the arterial lining, which increases the risk of blocking the artery again. We believe that virtual endoscopy, as a unique visualisation technique of presenting the intraluminal appearance of arterial wall as shown in our report, can be used as a valuable tool to identify any intimal changes of coronary artery due to tissue overgrowth before it leads to in-stent restenosis or thrombosis. With the aid of virtual endoscopy visualisation, this might then influence the time period needed for antiplatelet therapy, especially if further major open surgery is required. The potential applications of virtual endoscopy and stereoscopic visualisation in coronary stenting lie in the following aspects based on our report:

- Follow-up of coronary stenting (especially drug-eluting stent) with regard to the appearance of intraluminal wall with the aim of identifying the artery wall changes by use of virtual endoscopy;
- Confirmation of suspected in-stent re-stenosis in any section of the coronary artery branch with aid of virtual endoscopy;
- Enhancement of endovascular specialists’ understanding of the relationship between coronary stents and coronary arteries and appreciation of complex anatomic structures with the aid of 3D stereoscopic visualisation.
